# Time Series Analysis of Onchocerciasis Data from Mexico: A Trend towards Elimination

**DOI:** 10.1371/journal.pntd.0002033

**Published:** 2013-02-14

**Authors:** Edgar E. Lara-Ramírez, Mario A. Rodríguez-Pérez, Miguel A. Pérez-Rodríguez, Monsuru A. Adeleke, María E. Orozco-Algarra, Juan I. Arrendondo-Jiménez, Xianwu Guo

**Affiliations:** 1 Centro de Biotecnología Genómica, Instituto Politécnico Nacional, Reynosa, Tamaulipas, México; 2 Public Health Entomology and Parasitology Unit, Department of Biological Sciences, Osun State University, Osogbo, Osun, Nigeria; 3 Centro Nacional de Vigilancia Epidemiológica y Control de Enfermedades, Secretaria de Salud, México Distrito Federal, México; Imperial College London, Faculty of Medicine, School of Public Health, United Kingdom

## Abstract

**Background:**

In Latin America, there are 13 geographically isolated endemic foci distributed among Mexico, Guatemala, Colombia, Venezuela, Brazil and Ecuador. The communities of the three endemic foci found within Mexico have been receiving ivermectin treatment since 1989. In this study, we predicted the trend of occurrence of cases in Mexico by applying time series analysis to monthly onchocerciasis data reported by the Mexican Secretariat of Health between 1988 and 2011 using the software R.

**Results:**

A total of 15,584 cases were reported in Mexico from 1988 to 2011. The data of onchocerciasis cases are mainly from the main endemic foci of Chiapas and Oaxaca. The last case in Oaxaca was reported in 1998, but new cases were reported in the Chiapas foci up to 2011. Time series analysis performed for the foci in Mexico showed a decreasing trend of the disease over time. The best-fitted models with the smallest Akaike Information Criterion (AIC) were Auto-Regressive Integrated Moving Average (ARIMA) models, which were used to predict the tendency of onchocerciasis cases for two years ahead. According to the ARIMA models predictions, the cases in very low number (below 1) are expected for the disease between 2012 and 2013 in Chiapas, the last endemic region in Mexico.

**Conclusion:**

The endemic regions of Mexico evolved from high onchocerciasis-endemic states to the interruption of transmission due to the strategies followed by the MSH, based on treatment with ivermectin. The extremely low level of expected cases as predicted by ARIMA models for the next two years suggest that the onchocerciasis is being eliminated in Mexico. To our knowledge, it is the first study utilizing time series for predicting case dynamics of onchocerciasis, which could be used as a benchmark during monitoring and post-treatment surveillance.

## Author Summary

Mexico is one of the countries where human onchocerciasis (river blindness) can be found in Latin America. In 1989, the onchocerciasis program in Mexico started the treatment with ivermectin only for symptomatic individuals and then mass distribution of ivermectin was initiated for all eligible residents from 1994, either annually, twice or four times a year in endemic foci, coordinated by Mexican Secretariat of Health (MSH). In our study, we used a statistical method to analyse the cases of the disease reported by MSH from 1988 to 2011. The analysis showed that the cases of the disease have marginally decreased since 1999. The results also predicted an extremely low number (absence) of cases between 2012 and 2013 in the Chiapas region, the last endemic area, suggesting that disease is on a trend towards elimination in Mexico. Meanwhile, it could provide a benchmark for surveillance after mass treatment has been halted in 2012.

## Introduction

Human onchocerciasis is caused by the filarial worm *Onchocerca volvulus*, which is transmitted by the bites of blackflies of *Simulium* species [1]. The symptomatology of onchocerciasis disease is characterized by clinical manifestations such as onchocerca skin diseases, onchocercomata, lymphadenopathy, and ocular lesions, including the irremediable terminal effect of blindness [2]. Onchocerciasis is the major cause of blindness and dermatitis in endemic areas, and it remains as an important public health problem in Africa. In Latin America, there are six countries (Brazil, Colombia, Ecuador, Venezuela, Guatemala, and Mexico) with scattered and small endemic onchocerciasis foci, where a population of 470, 222 individuals is currently estimated to be at risk [3].

The discovery of onchocerciasis in America was in 1915 by Rodolfo Robles in Guatemala; hence it was first named as Robles's disease. In Mexico, the first cases of onchocerciasis were documented in 1923 in Chiapas, originated as a consequence of active seasonal migration of coffee workers from the endemic areas between Guatemala and Mexico. The regions in Chiapas and Oaxaca of Mexico are associated with the presence of abundant vector populations [4]. The focus of Oaxaca and Northern Chiapas represented the expansion of onchocerciasis from Southern Mexico or Guatemala [5].

The onchocerciasis control program in Mexico was first established in 1930 and has worked continuously up to date. During 1930–1946, a sporadic larval vector control campaign using Creolin was carried out to eliminate vector populations from breeding sites together with nodulectomy (removal of nodules) campaigns [6]. The administration of diethylcarbamazine (DEC) began in 1947, followed by a sporadic application of DDT to eliminate the vector populations in 1952. In 1990, DEC was supplanted by ivermectin (Mectizan; Merck & Co., Inc., Whitehouse Station, NJ) [4]. In 1992, the Onchocerciasis Elimination Program for the Americas (OEPA) was launched [7], and has successfully coordinated the efforts of the affected countries in Latin America. In 1989, the onchocerciasis program in Mexico started the treatment with ivermectin only for symptomatic individuals [4]. Later in 1997, ivermectin distribution was implemented twice a year for most eligible residents from all at-risk communities, followed by the distribution four times a year in the Chiapas foci as from 2003 upward, which was a successful strategy to accelerate the interruption of the parasite transmission [8]. As OEPA is preparing to wind up activities as from 2012, it is important to predict the possibility of future occurrence of new cases of the disease because there still exists the possibility of recrudesce due to the existence of potential infected vector population or multiple vectors [9].

The time series analysis has been applied in the field of epidemiological research on infectious diseases for the prediction of epidemiological spread tendency, which provided valuable information for making decisions in the control of such diseases [10]–[13]. For instance, the ARIMA models [14] as well as Seasonal Auto-Regressive Integrated Moving Average (SARIMA) [15] models were used to analyze time series data containing ordinary or seasonal trends of dengue cases to develop a forecasting model in endemic areas of Rio de Janeiro, Brazil and French West Indies, Guadeloupe respectively.

The univariate Auto-Regressive Integrated Moving Average (ARIMA) models are a kind of time series analysis for forecasting a time series data [16]. ARIMA models need a stationary series, that is, the mean and variances of the series are independent of time. Stationarity can be accomplished by data transformations and differencing. Once the series is stationary, it is fine-tuned through adding auto-regressive (AR) orders (lags of the differenced series) and/or moving average (MA) orders (lags of the forecast errors) as needed to remove any last hints of autocorrelation from the forecast errors. The usefulness of ARIMA models resides mostly in providing an estimate of the variability to be expected among future observations and depends on past values and random errors [10].

Herein, the cases of onchocerciasis reported by the Mexican Secretariat of Health (MSH) during the past two decades were analyzed using time series analysis. We adopted the ARIMA approach for describing the case dynamics of onchocerciasis in the endemic foci and predicted the tendency of occurrence of onchocerciasis cases in the immediate future.

## Methods

### Dataset

The official norm NOM-032-SSA2-2002 of MSH has defined that a case of onchocerciasis should comply with at least one of with the following requirements: demonstration of microfilariae through microscopic examination of superficial skin snips, identification of adult worms by removing nodules, observation of microfilariae in the cornea and anterior chamber of the eye, positive PCR and hybridization from skin snips or nodules. The individual should also present typical clinical manifestations of the disease, and inhabit or have resided in areas of active transmission. Monthly data of onchocerciasis cases between 1988 and 2010 were obtained from the MSH web site (http://www.dgepi.salud.gob.mx/anuario). Preliminary information on cases in 2011 was obtained from the weekly bulletin web site of MSH (http://www.dgepi.salud.gob.mx/boletin/).

### Time Series Analysis

Time series analysis for identifying significant predictors as well as for forecasting monthly onchocerciasis cases were carried out using the statistical analysis ARIMA model. The data in 1990 for Oaxaca and in 2001 for Chiapas was not available. The cases of onchocerciasis in Chiapas and Oaxaca from 1988 to 1993 were recorded every two months, which could result in data bias (one month with 0 cases after one month with data). Considering that the month without data does not indicate no case occurrence but the cases not reported, and then the cases of that month accumulated in the data of next month, we thus decided to adjust the data by assigning the half part of cases of a month to the previous zero-case month. Because of disease control activities (ivermectin distribution), cases of infection have been greatly reduced, giving rise to an abundance of zeros in the monthly case data. It needs to stabilize the variance of the series before seeking the best model that fits each dataset. The square root (sqrt) transformation was applied to stationarize our datasets. After stabilizing the variance, the descriptive method procedure was performed for plotting the onchocerciasis data through the autocorrelation function (ACF) and partial autocorrelation function (PACF) to identify the order of differentiation as well seasonal and non-seasonal effects. The residuals of the models fitted were inspected with the ACF and PACF plots and further verified with the Ljung-Box test. The best ARIMA model was selected for analysis according to the lowest Akaike Information Criterion (AIC). The ARIMA models were represented by the form as (p, d, q) (P, D, Q)S, where p is the order of auto-regression, d is the order of differencing (or integration), and q is the order of moving-average for non-seasonal series. P, D, Q are their seasonal counterparts, and S is the seasonal period. If the parameters p and q or P and Q are together present in the non-seasonal or seasonal series, the model was termed as mixed ARIMA model. We estimated the parameters of ARIMA models with the “arima” function implemented in the software R [17] that compute the exact likelihood via a state-space representation of the ARIMA process by using the Kalman filter [18], [19], “skipping” the missing observations in the computations, obtaining the maximum likelihood estimators of the model parameters. The model's fitted values were also graphically compared with the observed data. The fitted model was adopted to out-of-sample predict onchocerciasis cases for the next two years in the foci using the one-step ahead approach, that is, a forecast generated for the next observation only. For example, as the observed value for January 1998 was obtained in Oaxaca region, the data were updated to January 1998, re-estimated the parameters of the ARIMA model, and computed the next 1-step ahead predicted value, February 1998. This process was continued until the end of the year 1999. The software R (version 2.11.1) was used for all statistical analyses and graphic displays [17]. The automatic algorithms implemented in software R were also used to aid in the selection of the ARIMA models [20].

## Results

### Onchocerciasis Disease Patterns in Mexico

**Figure 1:**
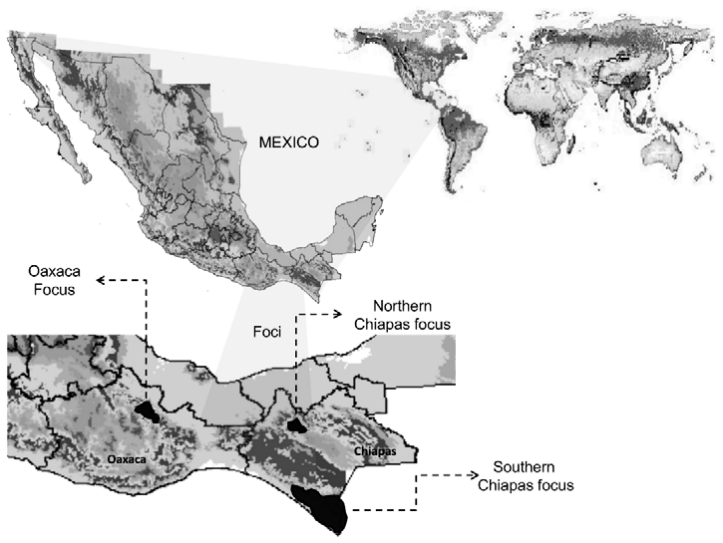
Map of the three endemic regions in the two southern Mexico states. The dark grey areas indicate the Oaxaca focus and the Northern and Southern Chiapas foci.

The cases of onchocerciasis in Mexico from 1988 to 2011 were summarized in Table S1. The total number onchocerciasis cases in Chiapas, Oaxaca and other regions of Mexico during 1988 to 2011 were 15,584 cases. The highest number of cases was in 1988 with 3,197 cases and afterwards the number decreased gradually, with the lowest number in 2010 with just 15 cases. The recorded cases were predominantly from two regions, Oaxaca and Chiapas (Figure 1), and some sporadic cases from other regions. In the Oaxaca focus, the total reported cases were 1,628; the number of cases was highest in 1991 and later decreased marginally. The last case in Oaxaca was recorded in 1998. Therefore, this disease had been successfully eliminated from the Oaxaca region. The Chiapas foci had a total of 13,849 cases reported. The case number remained high before 1990 and maintained a little lower level from 1991 to 1997, with the second peak in 1994. Then the recorded cases stably reduced, until 12 cases in 2011. There were 107 cases reported in other states (mainly Northern Mexico) during 1988–1993, 2005–2007, and 2009–2011, which were imported cases of onchocerciasis according to case definition of MSH.

### Time Series Analysis

**Figure 2:**
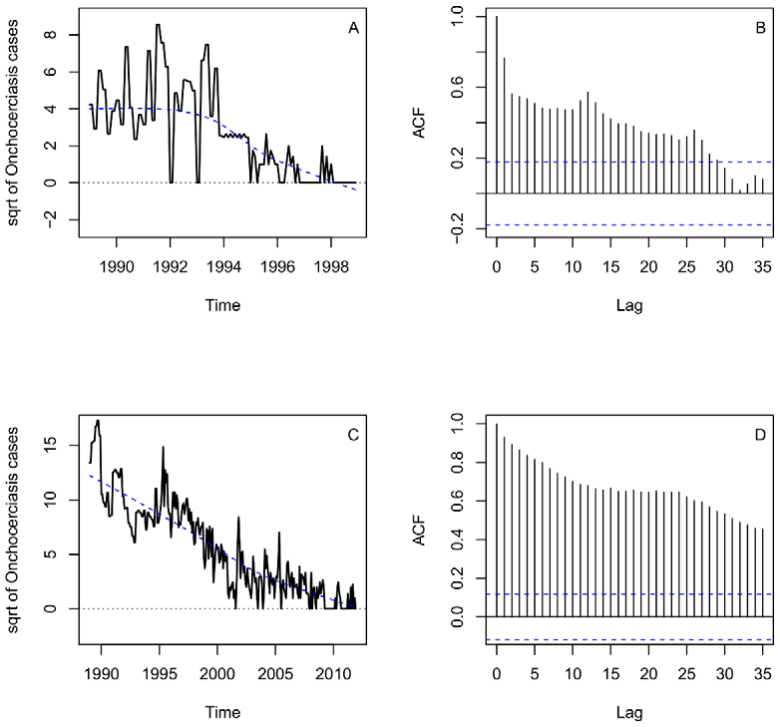
Time series profile and ACF plot for the data from Oaxaca and Chiapas. A and C) Time series profile of the square root values of onchocerciasis cases in Oaxaca and Chiapas. Dashed blue line indicates the trend of onchocerciasis series. B and D) Autocorrelation function (ACF) of onchocerciasis cases from Oaxaca and Chiapas. The x-axis represents the number of lags. Dashed blue line indicates 95% confidence interval.

**Figure 3:**
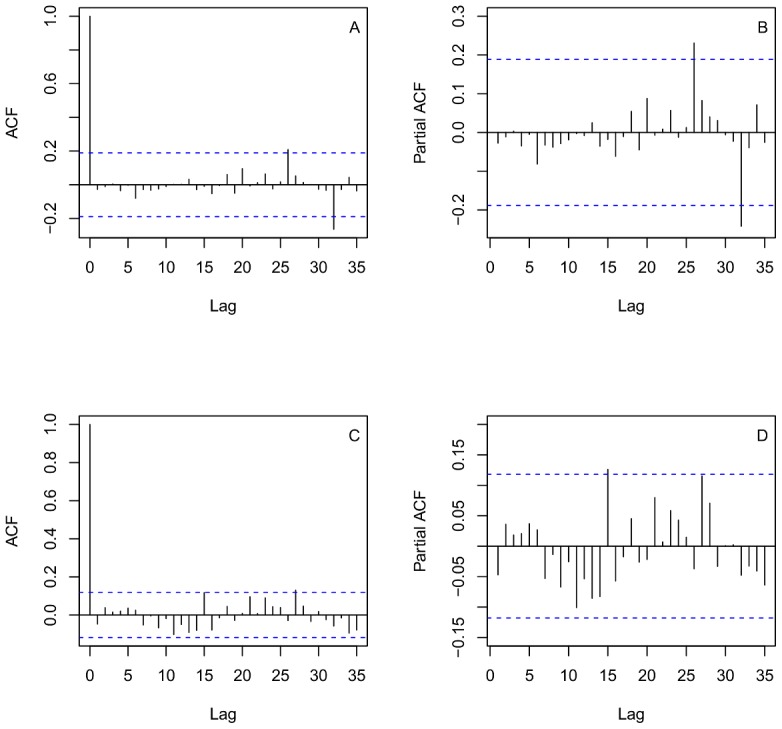
ACF and PACF plots of the residuals for the fitted models. A and B) Autocorrelation function (ACF) and Partial ACF (PACF) plot of the residuals of the ARIMA (1,1,1)x(0,0,1)_12_ model fitted for Oaxaca. C and D) Autocorrelation function (ACF) and Partial ACF (PACF) plot of the residuals of the ARIMA (1,1,1)x(1,0,0)_12_ model fitted for Chiapas. The x-axis represents the number of lags. Dashed blue lines indicate 95% confidence interval.

**Figure 4:**
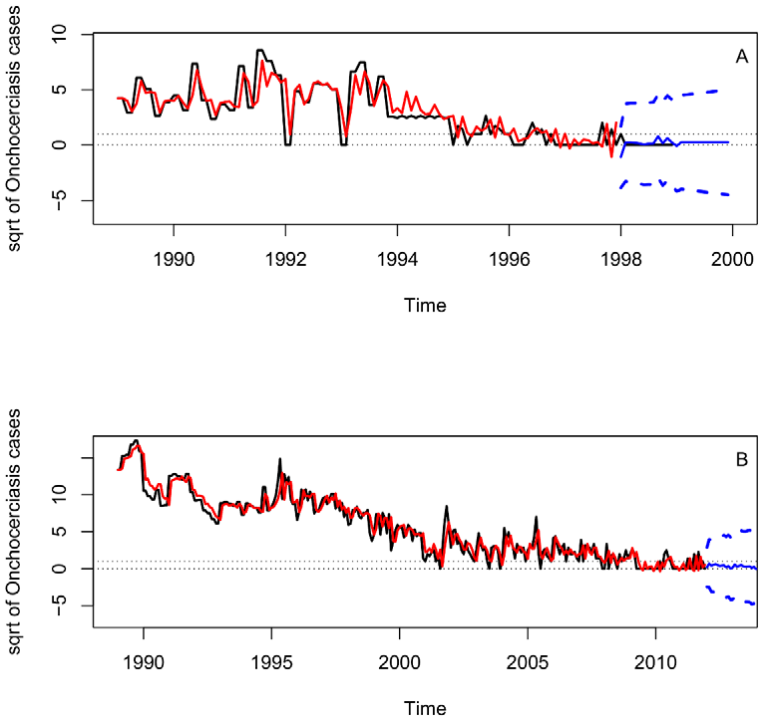
Time series profile for the observed data and for the fitted model. A). Black line: The square root curve of observed onchocerciasis cases in Oaxaca for the period 1988–1998. Solid red line: ARIMA (0,1,2)x(0,0,1)_12_ model's fitted values (1988–1997) and 1-step ahead predicted values (year 1998–1999) with their 95% prediction intervals (dashed blue line). B) Black line: The square root curve of observed onchocerciasis cases in Chiapas for the period 1988–2011. Solid red line: ARIMA (1,1,1)x(1,0,1)_12_ model's fitted values (1988–2011) and 1-step ahead predicted values (year 2012–2013) with their 95% prediction intervals (dashed blue lines).

In Oaxaca, there were no reported cases since 1999. This observation gave a good example for us to test if the Time Series Analysis describes well the dynamics of infection cases and predicted the approximate time of disease elimination in Oaxaca. The plot of sqrt-transformed onchocerciasis cases for Oaxaca showed a decreased trend since 1990 (Figure 2 A). The plot of ACF has positive autocorrelations out to a high number of lags, suggesting a nonstationary time series and a need for differencing (Figure 2 B). After stabilizing the series with the first order difference, we inspected the ACF and PACF plots, which suggests that non-seasonal and seasonal parameters are needed in the model (Figure S1 A, B). The negative ACF cutoff at lag 2, associated with the slow decay of PACF at lags 2–3 suggests that MA orders are needed in the model but the positive correlation at lag 12 associated with the PACF cutoff at lag 12 suggests that non-seasonal AR orders could be also added. In addition, the negative ACF cutoff at lag 32 associated with the slow decay of PACF at lags 28–31 suggested seasonal MA orders. Consequently, we fitted several ARIMA models with different Auto-Regressive orders, AR (p,P) and Moving Average orders, MA(q,Q), and excluded any models in which the residuals were not significant and had high AIC values. Thus, the best-fit model for Oaxaca was a mixed ARIMA (0,1,2)×(0,0,1)_12_ (AIC = 388.28) seasonal non-stationary model. All coefficients of ARIMA models for Oaxaca were significant (Table 1). The plots ACF and PACF of the residuals were almost located within the confidence limits (Figure 3 A, B). The Ljung–Box statistic test did not reject the null hypothesis of independence in the residuals time series (P value = 0.93). The model plot that fitted actual plot of dynamics of case data was shown in Figure 4 A. This model was then adopted for two-years-ahead prediction using the 1-step ahead approach. The forecast values for Oaxaca, showed a markedly decreasing trend and zero cases would occur from January 1998 to December 1999 (Figure 4 A), corresponding to the fact that the last case was reported from Oaxaca in 1998.

**Table 1:** Estimate parameters of the ARIMA models.

Region	ARIMA Model	Parameter	Coefficient	Stnd. Error	t statistic	P-value
Oaxaca	(0,1,2)×(0,0,1)_12_	MA(1)	−0.2235	0.0867	−2.57785467	2.48e^−03^
		MA(2)	−0.5917	0.0855	−6.92046784	1.11e^−12^
		SMA(1)	0.286	0.1015	2.81773399	1.21e^−03^
Chiapas	(1,1,1)×(1,0,1)_12_	AR(1)	0.5862	0.078	7.515384615	1.37e^−14^
		MA(1)	−0.8974	0.0432	−20.7731481	0.00e^+00^
		SAR(1)	0.8675	0.0841	10.31510107	0.00e^+00^
		SMA(1)	−0.746	0.1149	−6.49260226	2.15e^−11^

**ARIMA**. Auto-Regressive Integrated Moving Average. **AR.** Auto-Regressive. **MA.** Moving Average. **SMA.** Seasonal Moving average. **SAR.** Seasonal Auto-Regressive.

The predicted result that matches the observations in Oaxaca focus allows us to apply the same methodology to the Chiapas foci. Figure 2C showed the time series profile from 1988–2011 of onchocerciasis cases with the sqrt-transformation. As in Oaxaca, the plot of ACF showed a need for differencing because of a slow decay of positive autocorrelations out to a high number of lags (Figure 2 D). The ACF and PACF plots produced with the first order difference also suggest that non-seasonal and seasonal parameters are needed in the model (Figure S1 C, D). The negative ACF cutoff at lag 1, associated with the slow decay of PACF at lags 1–5, suggests non-seasonal MA orders. The positive ACF at lags 12 and 15 associated with the PACF sharp cutoffs at lags 12 and 15 also suggests that non-seasonal and seasonal AR orders could be added. Then, several ARIMA models with different AR and MA orders were fitted, excluding any models in which the residuals exhibited higher autocorrelation, non-significant coefficients and high AIC values. The best-fitted model obtained for Chiapas was a mixed ARIMA (1,1,1)×(1,0,1)_12_ (AIC = 960.77). All the coefficients of the ARIMA model for Chiapas were significant (Table 1). The plots ACF and PACF of the residuals show no remaining temporal correlation (Figure 3 C, D). The Ljung–Box statistic test did not reject the null hypothesis of independence in the residuals time series (P value = 0.34). Graphically the best-fitted model followed closely the decreasing trend of the observed series in Chiapas (Figure 4 B). The model was then used for two-years-ahead prediction using the 1-step ahead approach. It showed that the cases would continuously and markedly decrease in the recent years and the annual zero case could occur at the period from January of 2012 to December of 2013 in the Chiapas foci (Figure 4 B).

## Discussion

The key aspect in the control of onchocerciasis disease in Latin America is the treatment with the drug ivermectin available to all the people at risk [4], [8], [21]–[24]. The onchocerciasis program in Mexico began treatment with ivermectin in 1989, initially treating only symptomatic individuals in hyperendemic communities [4]. In 1994, annual mass ivermectin treatment to eligible residents (i.e., those who were 5 years older and who had resided in the endemic community) in the at-risk communities was initiated. From 1997, the strategy was modified to provide mass treatments twice a year to every eligible resident in the at-risk communities [8]. In Oaxaca, the new cases had been controlled effectively and the last case occurred in 1998. The situation in Chiapas is more complicated. It needs to note that the infection cases in Chiapas maintained a high platform before 1997 and there was a marked reduction from 953 cases in 1996 to 573 cases in 1997 and remained on a decreasing trend up to date.

In 2003, the biannual treatment strategy was modified in the majority of the formerly hyperendemic communities of Southern Chiapas focus by increasing treatment frequency to four times a year in order to accelerate the interruption of parasite transmission [25]. After this modification, the incidence was maintained at a low level (<100 cases) and reduced stably until the 12 cases in 2011. Thus, this observation indicates treatment of endemic communities with regimental distribution of Mectizan was a key to the elimination of onchocerciasis in Mexico, which is in agreement with a recent study [6]. At present, the transmission of onchocerciasis in Oaxaca and the Northern Chiapas has been eliminated, but the transmission was only interrupted in the Southern Chiapas focus as recently declared by OEPA [26]. Ivermectin treatment has been halted in the Southern Chiapas focus in 2012. Thus, all foci in Mexico are under epidemiological surveillance post-treatment which is within the fourth phase for certification of the elimination of onchocerciasis.

The above description shows that the elimination of onchocerciasis in humans is an arduous task. The evaluation of the current state and the prediction of future situations are germane for evaluating epidemiological patterns. Several mathematical models have been developed to simulate the onchocerciasis future in specific endemic zones [27]–[29]. These methods take into account mainly: the life cycle of the parasite, the type of treatment, the phenotypic characteristics of the vector, the microfilarial load in the skin, and the biting rate of the vectors. Two onchocerciasis transmission models were predominant in use that incorporated some of the above-mentioned variables [28], [30]–[32]. These mathematical models were applied to explore the epidemiological consequences and the effects of control interventions on the parasite population dynamics. One of the statistical analyses useful to make predictions is the time series analysis that has been used to study vector-borne diseases [15], [33], [34]. In the present study, the ARIMA models based on statistical concepts were fitted to onchocerciasis data collected from the endemic regions to predict the cases for the coming years. The Oaxaca focus has recorded the systematic data and there is no case since 1999, which provided a good example for practicing the method on describing the variation of cases in the focus and predicting the annual absence of infection case. A mixed ARIMA model was fitted to imitate the case variation data and predicted the absence of cases between 1998–1999, which coincides with the observed data, suggesting that the method is reliable. We then employed the similar ARIMA method for data from Chiapas, in which the mixed ARIMA model fitted well the observed data. Thus, this methodology could be considered to apply in other regions as the surveillance system for onchocerciasis. On the other hand, one goal of the onchocerciasis program in Mexico was to interrupt transmission of the parasite by the year 2012 [4]. Our model predicted that the values less than 1 case annually were located in the years 2012–2013. According to the World Health Organization (WHO) [35], [36] and OEPA [37] criteria to declare a place free of onchocerciasis, a reduction of new infections to an incidence rate of less than one new case per 1,000 individuals (<0.1%) and an absence, or near absence, of infective-stage larvae of *O. volvulus* in the vector population (i.e., a rate of less than one infective fly per 1,000 parous flies) must be documented in such area. If the current trend of onchocerciasis cases is sustained, the declaration of onchocerciasis elimination in Mexico would be in the not far future.

In the present study, sqrt transformation was chosen for stationarizing the series. Actually, Anscombe transform [38] (Figure S2) and the natural *log* (*ln*) (Figure S3) have been also tried. As approaching to elimination, cases of infection have been greatly reduced, giving rise to an abundance of zeros in the monthly case data. With the application of *ln*-transformation (the most common use to stabilize the variance), we cannot use the number “1” to replace the zero in the dataset, as done in the previous report [38], because the model will consider it as one real infection case and the cases could never be less than 1 in the prediction. Thus, log-transformations of the data were performed by adding a constant (<1) to the entire dataset. The results, based on the datasets transformed respectively by each of the three methods, were evaluated by the root-mean-square of the errors (RMSE). The RMSE values with the application of sqrt or Anscombe transform were similar and much smaller than with *ln*- transformation (Table S2), indicating that both sqrt and Anscombe transform are better than *ln*- transformation, which corresponds to the comment that logarithmic transformation is generally not recommended to apply to count dataset [39], particularly to small count dataset. Although there is almost no difference between sqrt and Anscombe transform in present study, sqrt could be more practical way to stationarize such datasets because sqrt is much easier to operate.

One phenomenon needs to be mentioned. The predicted plot in both Oaxaca and Chiapas showed that all the data in the coming years are less than 1 case but with 95% prediction interval beyond 1 case. A prediction interval is always wider than a confidence interval because it is not only related to the value of the population mean, but also the data scatter. When it approaches the elimination of disease, the number of cases show as 0, 1, or >1, not as continuous data less than 1 but approaching to 0. It means that as much closer to elimination, the distribution data become much more discretized. In this situation, it could be difficult to expect the prediction of annual cases less than 1 with the 95% prediction intervals within one case, even though the zero cases were treated as 0.1. On the other hand, our test for the focus in Oaxaca demonstrated our prediction is acceptable despite of the 95% prediction intervals beyond 1 case. This phenomenon mentioned here could be common for times series analysis as approaching to elimination of diseases. Our analysis thus provided a good reference for such prediction of similar diseases.

We realize that the present model is adopted for predicting the cases under the same situation in the recent future. Since 2012, the mass treatments with ivermectin have been halted. Does it mean our prediction does not work? If ivermectin is the principal reason leading to reduction of cases and the transmission has been really interrupted, it is very possible that the tendency of case occurrence could keep reduction until to zero case. In other words, the mass treatments with ivermectin still keep the influence (the consequence of the treatment) and our prediction model has the condition to work. Otherwise, the mass treatments with ivermectin could need to be re-continued. To this sense, the application of this prediction model could be used as a benchmark during monitoring and surveillance after mass treatment has been withdrawn.

We are aware of the possible limitations of the present study. The data used in the current study rely on total clinical cases of onchocerciasis reported by the surveillance system of MSH, which may underestimate the true number of cases as earlier posited by various researchers [38], [40], [41]. Another limitation is the heterogeneity of the data used that could affect the time series analysis. However, the application of this method in Oaxaca focus indicated that our data analysis was adequate. Therefore, the time series analysis applied herein is acceptable.

In conclusion, onchocerciasis in Mexico was a serious public health problem in the past. ARIMA models predicted an extremely low (zero) expected cases of onchocerciasis for the next two years, implying that onchocerciasis is being eliminated. These results showed that time series analysis could be a practical method for predicting onchocerciasis case tendencies and could be used as a benchmark for monitoring and surveillance on the post ivermectin-mass-treatment duration. To our knowledge, it is the first study utilizing time series analysis for predicting the case dynamics of onchocerciasis.

## Supporting Information

Checklist S1 **STROBE checklist.**
(DOC)

Figure S1 **ACF and PACF plots produced with the first order difference.** A and B) Autocorrelation function (ACF) and Partial ACF (PACF) plot for Oaxaca. C and D) Autocorrelation function (ACF) and Partial ACF (PACF) plot for Chiapas. The x-axis represents the number of lags. Dashed blue lines indicate 95% confidence interval.
(TIF)

Figure S2 **Time series profile for the observed data and for the fitted model.** A). Black line: The Anscombe transform curve of observed onchocerciasis cases in Oaxaca for the period 1988–1998. Solid red line: ARIMA (0,1,2)x(0,0,1)_12_ model's fitted values (1988–1997) and 1-step ahead predicted values (year 1998–1999) with their 95% prediction intervals (dashed blue line). B) Black line: The Anscombe transform curve of observed onchocerciasis cases in Chiapas for the period 1988–2011. Solid red line: ARIMA (1,1,1)x(1,0,1)_12_ model's fitted values (1988–2011) and 1-step ahead predicted values (year 2012–2013) with their 95% prediction intervals (dashed blue lines).
(TIF)

Figure S3 **Time series profile for the observed data and for the fitted model.** A). Black line: The natural *ln* curve of observed onchocerciasis cases in Oaxaca for the period 1988–1998. Solid red line: ARIMA (1,1,1)x(0,0,1)_12_ model's fitted values (1988–1997) and 1-step ahead predicted values (year 1998–1999) with their 95% prediction intervals (dashed blue line). B) Black line: The natural *ln* transform curve of observed onchocerciasis cases in Chiapas for the period 1988–2011. Solid red line: ARIMA (1,1,1)x(1,0,0)_12_ model's fitted values (1988–2011) and 1-step ahead predicted values (year 2012–2013) with their 95% prediction intervals (dashed blue lines).
(TIF)

Table S1 **Annual onchocerciasis cases in Mexico from 1988–2011.**
(DOC)

Table S2 **The values of RMSE applied for evaluating the different data transformation methods.**
(DOC)
