# Supplementary material for: Time Series Analysis of Onchocerciasis Data from Mexico: A Trend towards Elimination
Source: PLoS Negl Trop Dis. 2013 Feb 14;7(2):e2033. doi: 10.1371/journal.pntd.0002033 (PMC3573083; doi:10.1371/journal.pntd.0002033)
Supplement: Checklist S1 — STROBE checklist. (DOC) [file pntd.0002033.s001.doc]

STROBE Statement

|  | Item No |  |
| --- | --- | --- |
| **Title and abstract** | 1 | *Time Series Analysis of Onchocerciasis Data from Mexico.* |
|  |  | A total of 15,584 cases were reported in Mexico from 1988 to 2011. The data of onchocerciasis cases are mainly from the main endemic foci of Chiapas and Oaxaca. The last case in Oaxaca was reported in 1998; but new cases were reported in the Chiapas foci up to 2011. Time series analysis performed for the foci in Mexico showed a decreasing trend of the disease over time. The best-fitted models with the smallest Akaike Information Criterion (AIC) were Auto-Regressive Integrated Moving Average (ARIMA) models, which were used to predict the tendency of onchocerciasis cases for two years ahead. According to the ARIMA models predictions, the cases in very low number (below 1) are expected for the disease between 2012 and 2013 in Chiapas, the last endemic region in Mexico. To our knowledge, it is the first study utilizing time series for predicting case dynamics of onchocerciasis, which could be used as a benchmark during monitoring and surveillance after mass treatment has been withdrawn. |
| Introduction | | |
| Background/rationale | 2 | *In Latin America, there are 13 geographically isolated endemic foci distributed among Mexico, Guatemala, Colombia, Venezuela, Brazil and Ecuador, where a population of 470, 222 individuals is currently estimated to be at risk. In 1990, DEC was supplanted by ivermectin (Mectizan; Merck & Co., Inc., Whitehouse Station, NJ). In 1992, the Onchocerciasis Elimination Program for the Americas (OEPA) was launched, and has successfully coordinated the efforts of the affected countries in Latin America. The communities of the three endemic foci in Mexico have been receiving ivermectin treatment since 1989. Later in 1997, ivermectin distribution was implemented twice a year for most eligible residents from all at-risk communities, followed by the distribution four times a year in the Chiapas foci as from 2003 upward. The time series analysis has been applied in the field of epidemiological research on infectious diseases for the prediction of epidemiological spread tendency, which provided valuable information for making decisions in the control of such diseases. The univariate Auto-Regressive Integrated Moving Average (ARIMA) models are a kind of time series analysis for forecasting a time series data. In this study, we predicted the trend of occurrence of cases in Mexico by applying time series analysis to monthly onchocerciasis data reported by the Mexican Secretariat of Health (MSH) between 1988 and 2011 using the software R.* |
| Objectives | 3 | Apply the time series analysis to predict the case trend of onchocerciasis in Mexico, which could provide valuable information for the control of this disease. |
| Methods | | |
| Study design | 4 | *Using longitudinal retrospective to predict the case trend in Mexico*. |
| Setting | 5 | *Monthly data of onchocerciasis cases between 1988 and 2010 were obtained from the MSH web site (http: www.dgepi.salud.gob.mx/anuario). Preliminary information on cases in 2011 was obtained from the weekly bulletin web site of MSH (http://www.dgepi.salud.gob.mx/boletin/).* |
| Participants | 6 | (*a*) *Cohort study*—Give the eligibility criteria, and the sources and methods of selection of participants. Describe methods of follow-up  *Case-control study*—Give the eligibility criteria, and the sources and methods of case ascertainment and control selection. Give the rationale for the choice of cases and controls  *Cross-sectional study*—Give the eligibility criteria, and the sources and methods of selection of participants |
|  |  | (*b*) *Cohort study*—For matched studies, give matching criteria and number of exposed and unexposed  *Case-control study*—For matched studies, give matching criteria and the number of controls per case |
| Variables | 7 | *The official norm NOM-032-SSA2-2002 of MSH has defined that a case of onchocerciasis should comply, with at least one of with the following requirements: demonstration of microfilariae through microscopic examination of superficial skin snips, identification of adult worms by removing nodules, observation of microfilariae in the cornea and anterior chamber of the eye, positive PCR and hybridization from skin snips or nodules. The individual should also present typical clinical manifestations of the disease, and inhabit or have resided in areas of active transmission.* |
| Data sources/ measurement | 8* | *Monthly data of onchocerciasis cases between 1988 and 2010 were obtained from the MSH web site (http: www.dgepi.salud.gob.mx/anuario). Preliminary information on cases in 2011 was obtained from the weekly bulletin web site of MSH (http://www.dgepi.salud.gob.mx/boletin/).* |
| Bias | 9 | *The cases of onchocerciasis in Chiapas and Oaxaca from 1988 to 1993 were recorded every two months, which could result in data bias (one month with 0 cases after one month with data). Considering that the month without data does not indicate no case occurrence but the cases not reported, and then the cases of that month accumulated to be reported in the data of next month, we thus decide to adjust the data by assigning the half part of cases of a month to the previous zero-case month* |
| Study size | 10 | *A total of 15,584 cases reported in Mexico from 1988 to 2011 were employed in the present study****.*** |
| Quantitative variables | 11 | *Because of disease control activities (ivermectin distribution), cases of infection have been greatly reduced, giving rise to an abundance of zeros in the monthly case data. However, the zeros in the data affect making a log transformation (a step required to stationarize the series) in the analysis. We cannot use the number “1” to replace the zero, as done in some other report, because the model will consider it as one real infection case and the cases could never be less than 1 in the prediction. Thus, log-transformations of the data were performed, replacing zeros with an arbitrary (<1) constant.* |
| Statistical methods | 12 | *Time series analysis for identifying significant predictors as well as for forecasting monthly onchocerciasis cases were carried out using the statistical analysis ARIMA model. After stabilizing the variance by the sqrt-transformation of the series, the descriptive method procedure was performed for plotting the onchocerciasis data through the autocorrelation function (ACF) and partial autocorrelation function (PACF) to identify the order of differentiation as well seasonal and non-seasonal effects. The residuals of the models fitted were inspected with the ACF and PACF plots and further verified with the Ljung-Box test. The best ARIMA model was selected for analysis according to the lowest Akaike Information Criterion (AIC). The ARIMA models were represented by the form as (p, d, q) (P, D, Q)S, where p is the order of auto-regression, d is the order of differencing (or integration), and q is the order of moving-average for non-seasonal series. P, D, Q are their seasonal counterparts, and S is the seasonal period. If the parameters p and q or P and Q are together present in the non-seasonal or seasonal series, the model was termed as mixed ARIMA model. We estimated the parameters of ARIMA models with the “arima” function implemented in the software R that compute the exact likelihood via a state-space representation of the ARIMA process by using the Kalman filter, “skipping” the missing observations in the computations, obtaining the maximum likelihood estimators of the model parameters. The model’s fitted values were also graphically compared with the observed data. The fitted model was adopted to out-of-sample predict onchocerciasis cases for the next two years in the foci using the one-step ahead approach, that is, a forecast generated for the next observation only. For example, as the observed value for January 1998 was obtained in Oaxaca region, the data were updated to January 1998, re-estimated the parameters of the ARIMA model, and computed the next 1-step ahead predicted value, February 1998. This process was continued until the end of the year 1999. The software R (version 2.11.1) was used for all statistical analyses and graphic displays. The automatic algorithms implemented in software R were also used to aid in the selection of the ARIMA models.* |
|  |  | (*b*) Describe any methods used to examine subgroups and interactions |
|  |  | (*c*) *The cases of onchocerciasis in Chiapas and Oaxaca from 1988 to 1993 were recorded every two months, which could result in data bias (one month with 0 cases after one month with data). Considering that the month without data does not indicate no case occurrence but the cases not reported, and then the cases of that month accumulated to be reported in the data of next month, we thus decide to adjust the data by assigning the half part of cases of a month to the previous zero-case month* |
|  |  | (*d*) *Cohort study*—If applicable, explain how loss to follow-up was addressed  *Case-control study*—If applicable, explain how matching of cases and controls was addressed  *Cross-sectional study*—If applicable, describe analytical methods taking account of sampling strategy |
|  |  | (*e*) Describe any sensitivity analyses |

Continued on next page

| Results | | |
| --- | --- | --- |
| Participants | 13* | (a) Report numbers of individuals at each stage of study—eg numbers potentially eligible, examined for eligibility, confirmed eligible, included in the study, completing follow-up, and analysed |
|  |  | (b) Give reasons for non-participation at each stage |
|  |  | (c) Consider use of a flow diagram |
| Descriptive data | 14* | *The total number onchocerciasis cases in Chiapas, Oaxaca and other regions of Mexico during 1988 to 2011 were 15,584 cases. The highest number of cases was in 1988 with 3,197 cases and afterwards the number decreased gradually, with the lowest number in 2010 with just 15 cases. The recorded cases were predominantly from two regions, Oaxaca and Chiapas, and some sporadic cases from other regions. In the Oaxaca focus, the total reported cases were 1,628; the number of cases was highest in 1991 and later decreased marginally. The last case in Oaxaca was recorded in 1998. Therefore, this disease had been successfully eliminated from the Oaxaca region. The Chiapas foci had a total of 13,849 cases reported. The case number remained high before 1990 and maintained a little lower level from 1991 to 1997, with the second peak in 1994. Then the recorded cases stably reduced, until 12 cases in 2011. There were 107 cases reported in other states (mainly Northern Mexico) during 1988-1993, 2005-2007, and 2009-2011, which were imported cases of onchocerciasis according to case definition of MSH*. |
|  |  | (b) Indicate number of participants with missing data for each variable of interest |
|  |  | (c) *Cohort study*—Summarise follow-up time (eg, average and total amount) |
| Outcome data | 15* | *Cohort study*—Report numbers of outcome events or summary measures over time |
|  |  | *Case-control study—*Report numbers in each exposure category, or summary measures of exposure |
|  |  | *Cross-sectional study—*Report numbers of outcome events or summary measures |
| Main results | 16 | *In Oaxaca, there were no reported cases since 1999. This observation gave a good example for us to test if the Time Series Analysis describes well the dynamics of infection cases and predicted the approximate time of disease elimination in Oaxaca. We established a mixed ARIMA model (0,1,2) x (0,0,1)_12_ (AIC = 388.28) that well fitted the dynamics of observed case data. This model was then adopted for two-years-ahead prediction using the 1-step ahead approach. The forecast values for Oaxaca, showed a markedly decreasing trend and zero cases would occur from January 1998 to December 1999, corresponding to the fact that the last case was reported from Oaxaca in 1998.*  *The predicted result which matches the observations in Oaxaca focus allows us to apply the same methodology to the Chiapas foci. The best model obtained for Chiapas was a mixed ARIMA (1,1,1) x (1,0,1)_12_ (AIC = 960.67). The model was then used for two-years-ahead prediction using the 1-step ahead approach. It showed that the cases would continuously and markedly decrease in the recent years and the annual zero case could occur at the period from January of 2012 to December of 2013 in the Chiapas foci.* |
|  |  | (*b*) Report category boundaries when continuous variables were categorized |
|  |  | (*c*) If relevant, consider translating estimates of relative risk into absolute risk for a meaningful time period |
| Other analyses | 17 | Report other analyses done—eg analyses of subgroups and interactions, and sensitivity analyses |
| Discussion | | |
| Key results | 18 | *Our model predicted that the values less than one case annually were located in the years 2012- 2013. Thus, the extremely low level of expected cases as predicted by ARIMA models for the next two years suggest that the onchocerciasis is being eliminated in Mexico. Time series analysis can be used for the prediction of case trend of onchocerciasis, which could be considered to apply in other regions as the surveillance system for onchocerciasis. It is the first study utilizing time series for predicting case dynamics of onchocerciasis, which could be used as a benchmark during monitoring and surveillance after mass treatment has been withdrawn.* |
| Limitations | 19 | *The data used in the current study rely on total clinical cases of onchocerciasis reported by the surveillance system of MSH, which may underestimate the true number of cases as earlier posited by various researchers. Another limitation is the heterogeneity of the data used that could affect the time series analysis. However, the application of this method in Oaxaca focus indicated that our data analysis was adequate. Therefore, the time series analysis applied herein is acceptable* |
| Interpretation | 20 | *The onchocerciasis in Mexico was a serious public health problem in the past. ARIMA models predicted an extremely low (zero) expected cases of onchocerciasis for the next two years, implying that onchocerciasis is being eliminated. These results showed that time series analysis could be a practical method for predicting onchocerciasis case tendencies and could be used as a benchmark for monitoring and surveillance on the post ivermectin-mass-treatment duration. This is the first study utilizing time series analysis for predicting the case dynamics of onchocerciasis.* |
| Generalisability | 21 | *Time series analysis can be used for the prediction of case trend of onchocerciasis, which could be considered to apply in other regions as the surveillance system for onchocerciasis. It could be also used as a benchmark during monitoring and surveillance after mass treatment has been withdrawn.* |
| Other information | | |
| Funding | 22 | Give the source of funding and the role of the funders for the present study and, if applicable, for the original study on which the present article is based |

*Give information separately for cases and controls in case-control studies and, if applicable, for exposed and unexposed groups in cohort and cross-sectional studies.

**Note:** An Explanation and Elaboration article discusses each checklist item and gives methodological background and published examples of transparent reporting. The STROBE checklist is best used in conjunction with this article (freely available on the Web sites of PLoS Medicine at http://www.plosmedicine.org/, Annals of Internal Medicine at http://www.annals.org/, and Epidemiology at http://www.epidem.com/). Information on the STROBE Initiative is available at www.strobe-statement.org.
