# Supplementary figures and images for: Time Series Analysis of Onchocerciasis Data from Mexico: A Trend towards Elimination
Source: PLoS Negl Trop Dis. 2013 Feb 14;7(2):e2033. doi: 10.1371/journal.pntd.0002033 (PMC3573083; doi:10.1371/journal.pntd.0002033)

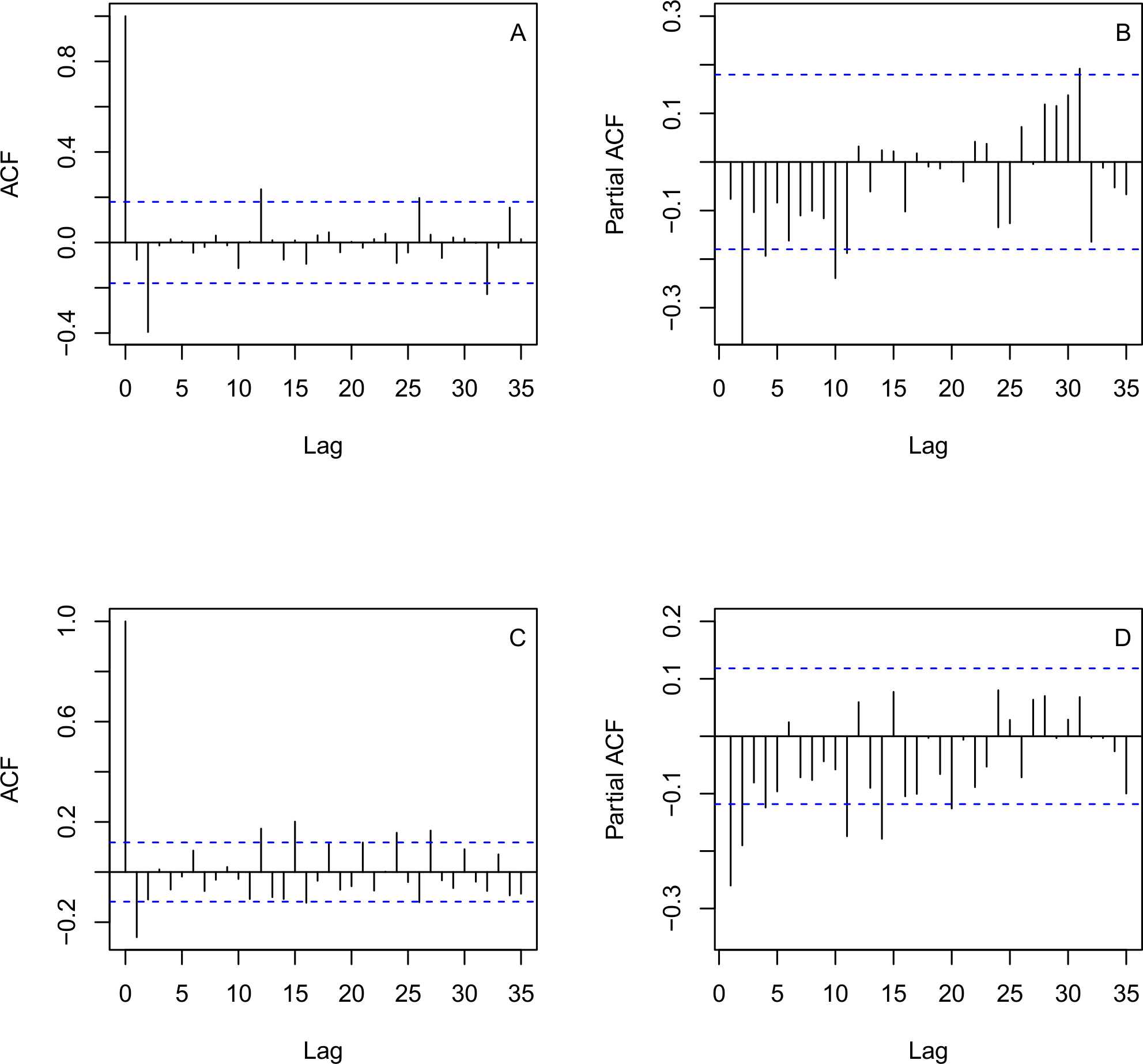

Supplement: Figure S1 — ACF and PACF plots produced with the first order difference. A and B) Autocorrelation function (ACF) and Partial ACF (PACF) plot for Oaxaca. C and D) Autocorrelation function (ACF) and Partial ACF (PACF) plot for Chiapas. The x-axis represents the number of lags. Dashed blue lines indicate 95% confidence interval. (TIF) [file pntd.0002033.s002.tif]

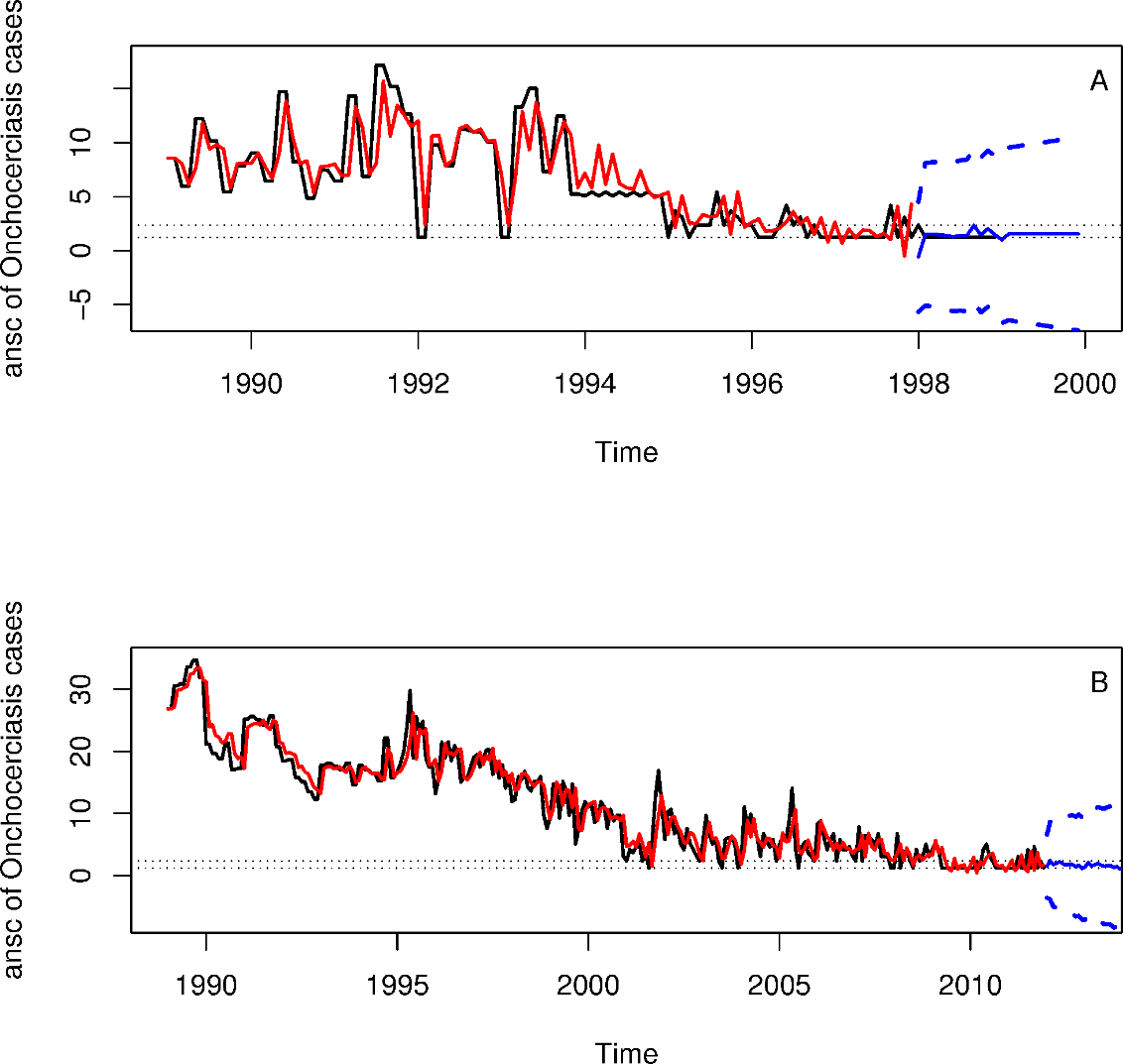

Supplement: Figure S2 — Time series profile for the observed data and for the fitted model. A). Black line: The Anscombe transform curve of observed onchocerciasis cases in Oaxaca for the period 1988–1998. Solid red line: ARIMA (0,1,2)x(0,0,1)12 model's fitted values (1988–1997) and 1-step ahead predicted values (year 1998–1999) with their 95% prediction intervals (dashed blue line). B) Black line: The Anscombe transform curve of observed onchocerciasis cases in Chiapas for the period 1988–2011. Solid red line: ARIMA (1,1,1)x(1,0,1)12 model's fitted values (1988–2011) and 1-step ahead predicted values (year 2012–2013) with their 95% prediction intervals (dashed blue lines). (TIF) [file pntd.0002033.s003.tif]

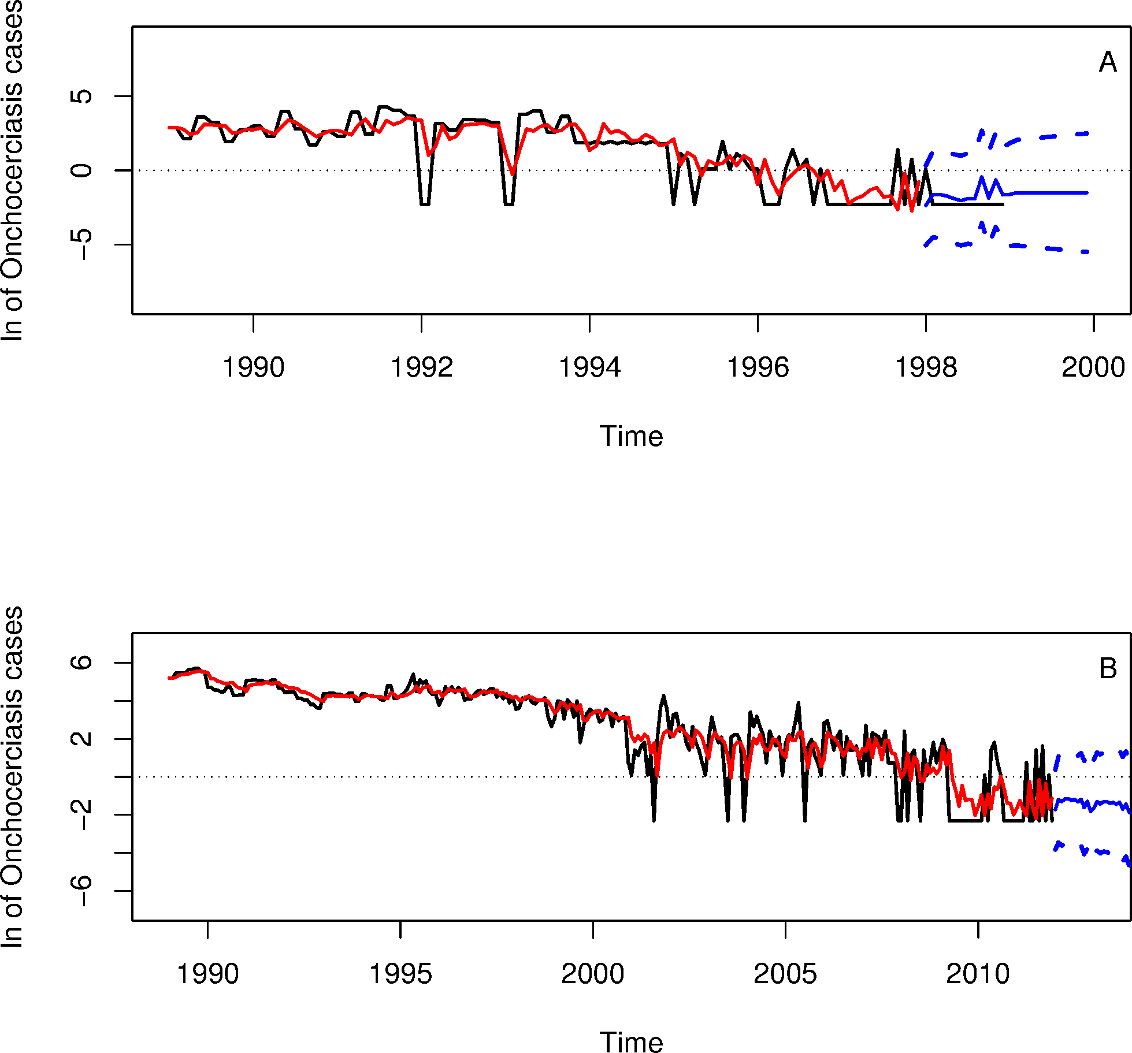

Supplement: Figure S3 — Time series profile for the observed data and for the fitted model. A). Black line: The natural ln curve of observed onchocerciasis cases in Oaxaca for the period 1988–1998. Solid red line: ARIMA (1,1,1)x(0,0,1)12 model's fitted values (1988–1997) and 1-step ahead predicted values (year 1998–1999) with their 95% prediction intervals (dashed blue line). B) Black line: The natural ln transform curve of observed onchocerciasis cases in Chiapas for the period 1988–2011. Solid red line: ARIMA (1,1,1)x(1,0,0)12 model's fitted values (1988–2011) and 1-step ahead predicted values (year 2012–2013) with their 95% prediction intervals (dashed blue lines). (TIF) [file pntd.0002033.s004.tif]
