# Supplementary material for: Time Series Analysis of Onchocerciasis Data from Mexico: A Trend towards Elimination
Source: PLoS Negl Trop Dis. 2013 Feb 14;7(2):e2033. doi: 10.1371/journal.pntd.0002033 (PMC3573083; doi:10.1371/journal.pntd.0002033)
Supplement: Table S1 — Annual onchocerciasis cases in Mexico from 1988–2011. (DOC) [file pntd.0002033.s005.doc]

**Table S1. Annual onchocerciasis cases in Mexico from 1988-2011**

|  | **Oaxaca** | **Chiapas** | **Other regions** | **Total** |
| --- | --- | --- | --- | --- |
| **Years** | **No. of Cases** | **No. of Cases** | **No. of Cases** | **No. of Cases** |
| 1988 | 222 | 2969 | 6 | 3197 |
| 1989 | 239 | 1110 | 46 | 1395 |
| 1990 | * | 1791 | 24 | 1815 |
| 1991 | 486 | 745 | 7 | 1238 |
| 1992 | 249 | 888 | 1 | 1138 |
| 1993 | 316 | 917 | 1 | 1234 |
| 1994 | 77 | 1393 | 0 | 1470 |
| 1995 | 22 | 967 | 0 | 989 |
| 1996 | 10 | 953 | 0 | 963 |
| 1997 | 6 | 573 | 0 | 579 |
| 1998 | 1 | 410 | 0 | 411 |
| 1999 | 0 | 274 | 0 | 274 |
| 2000 | 0 | 174 | 0 | 174 |
| 2002 | 0 | 125 | 0 | 125 |
| 2003 | 0 | 88 | 0 | 88 |
| 2004 | 0 | 126 | 0 | 126 |
| 2005 | 0 | 113 | 12 | 125 |
| 2006 | 0 | 92 | 2 | 94 |
| 2007 | 0 | 66 | 2 | 68 |
| 2008 | 0 | 34 | 0 | 34 |
| 2009 | 0 | 15 | 1 | 16 |
| 2010 | 0 | 14 | 1 | 15 |
| 2011 | 0 | 12 | 4 | 16 |
| Total cases | 1628 | 13849 | 107 | 15584 |

*. The data of 1990 for Oaxaca and of 2001 for Chiapas were not available
