# Supplementary material for: Time Series Analysis of Onchocerciasis Data from Mexico: A Trend towards Elimination
Source: PLoS Negl Trop Dis. 2013 Feb 14;7(2):e2033. doi: 10.1371/journal.pntd.0002033 (PMC3573083; doi:10.1371/journal.pntd.0002033)
Supplement: Table S2 — The values of RMSE applied for evaluating the different data transformation methods. (DOC) [file pntd.0002033.s006.doc]

**Table S2 The values of RMSE applied for evaluating the different data transformation methods.**

| **Regions** | **Transformation** | **RMSE** |
| --- | --- | --- |
| Oaxaca | sqrt | 11.78 |
|  | Ascombe | 11.62 |
|  | *ln* | 14.54 |
|  |  |  |
| Chiapas |  |  |
|  | sqrt | 20.77 |
|  | Ascombe | 20.70 |
|  | *ln* | 23.04 |
